# Low compliance contribute to insufficient Desmopressin response of primary monosymptomatic nocturnal enuresis and the role of voiding school

**DOI:** 10.1186/s12887-021-02714-z

**Published:** 2021-05-20

**Authors:** Zoran Radojicic, Sasa Milivojevic, Irena Koricanac, Jelena Milin Lazovic, Darko Laketic, Ognjen Radojicic, Natasa Milic

**Affiliations:** 1 University Children’s Hospital Belgrade, Belgrade, Serbia; 2 Institute for Medical Statistics and Informatics, Faculty of Medicine, University of Belgrade, Belgrade, Serbia; 3 Institute of anatomy “Niko Miljanic”, Faculty of medicine, Belgrade, Serbia; 4 Clinic for Gynecology and Obstetrics “Narodni Front”, Belgrade, Serbia; 5 Department of Internal Medicine, Mayo Clinic, Rochester, USA

**Keywords:** Primary Monosymptomatic nocturnal enuresis, Desmopressin, Compliance, Voiding school, Children

## Abstract

**Aims:**

To evaluate the impact of compliance on the therapeutic effects of Desmopressin, as well as the importance of establishing the voiding school for low-compliance children in primary monosymptomatic enuresis treatment.

**Methods:**

Eighty-nine patients with primary monosymptomatic enuresis treated with Desmopressin were observed during the 2017–2020 at University Children’s Hospital Belgrade, Serbia. The average patients age was 7.7 ± 2.4 years; 65 (73%) were boys and 24 (27%) % were girls. After the 3 months of Desmopressin treatment, the effect of therapy was evaluated according to the compliance. After the treatment, low-compliance patients and their parents were suggested to visit a voiding school.

**Results:**

A significant decrease in the median enuresis frequency was noticed during the Desmopressin treatment (25.0 (20.0–26.0) vs 10.0 (2.0–17.0) per month, before vs after treatment, respectively) (*p* < 0.001). Patients with low compliance had a poorer response to Desmopressin (*p* < 0.001). An median enuresis reduction in the good compliance group was 92.3% (86.7 -95%), while in the low compliance group was 28.6% (16.7–43.3%). After attending voiding school, there was a significant increase in compliance (*p* < 0.001), associated with an median percent decrease in enuresis of 84.0% (75.0–95.5%) (*p* < 0.001).

**Conclusion:**

Compliance considerably influences the beneficial effects of Desmopressin. Patients with poor therapeutic effects should be evaluated for compliance and introduced to voiding school.

## Introduction

Monosymptomatic nocturnal enuresis (MNE) is intermittent, uncontrolled sleep-time bedwetting without any signs or symptoms of lower urinary tract dysfunction in child after the age of five [1]. Considering that bedwetting may have major consequences on social and emotional well-being of a child, its treatment is essential [2]. In a view that nocturnal polyuria (NP) is a leading characteristics of MNE, Desmopressin is a treatment of choice [3, 4]. Although many children obtain only a partial response to Desmopressin, just few studies examined why. One of the reasons may be low compliance with Desmopressin [5]. Voiding school can be the introduced for therapy-resistant cases. Important interactions between children, parents and voiding school multidisciplinary team can improve children self-confidence and their social functioning [6, 7]. The aim of this study was to evaluate the impact of compliance with Desmopressin on its therapeutic effects, as well as the importance of establishing the voiding school for low-compliance children in primary MNE.

## Materials and method

A prospective cohort study, observing primary MNE patients, was carried out from 2017 to 2020 at the Urology Department of the University Children’s Hospital in Belgrade. A total of 124 patients were diagnosed with primary MNE, 96 met the inclusion criteria, and 89 patients agreed to participate in the study.

Detailed inclusion and exclusion criteria, as well as patient assessment criteria are published previously [8]. Briefly, patients with active urinary tract infection, non-mono symptomatic nocturnal enuresis (NMNE), bowel dysfunction (constipation/encopresis), endocrine, neurological and psychiatric disorders, hypertension, presence or history of renal disease, genitourinary or congenital abnormality, secondary enuresis and previous medical/alarm therapy for nocturnal enuresis were excluded from the study. NMNE was defined as increased/decreased voiding frequency, daytime incontinence, urgency, hesitancy, straining, a weak stream, intermittency, holding maneuvers, a feeling of incomplete emptying, postvoiding dribble and genital or lower urinary tract pain. Urine analysis, serum creatinine, blood urine nitrogen, sodium, potassium, and complete blood count, as well as ultrasound of kidney and urinary tract were performed if necessary. Finally, the study included only the primary MNE patients in whom solely nocturnal polyuria was diagnosed, i.e. the existence of a lower-capacity bladder was ruled out. All patients were newly diagnosed with primary MNE, were medical treatment naive, and did not respond to conservative modalities for 3 months at least. Regular bladder emptying before sleep, reduced higher fluid intake, avoiding dairy, protein-rich and heavy loads of salt products during 3–4 h before bedtime, voiding 4–7 times per day and drinking at least 1 l/day were considered as conservative modalities [9]. Number of wet nights in the month proceeding the treatment was determined for all patients.

Detailed treatment protocol is described elsewhere [8]. Patients were prescribed Desmopressin 0.2 mg daily per os, at least 1 h before bedtime orally. During the treatment period children were followed every 4 weeks. All patients and their parents were asked to keep a noctural enuresis calendar recording wet and dry nights, adherence to therapy and dietary recommendations. After the 3-month of Desmopressin therapy, its effect (the decrease in number of wet nights over the previous month) was evaluated according to compliance. Compliance was determined based on a diary, filled up over 7 days during the last week of Desmopressin treatment, with statements regarding: non-regular drug intake, improper dosage, arbitrary discontinuation of therapy, inproper drug administration, fluid intake after the drug intake, higher fluid intake in the evening than recommended, not performing voiding training and not adhering to diet instructions. The study included 89 consecutive patients with primary MNE prospectively followed. After Desmopressin treatment, study population was divided according to therapy compliance - on low and good compliance groups. Good compliance group included 35 patients who followed all instructions during treatment period i.e. were adherent with all instructions regarding Desmopressin intake and were adherent to all specified conservative measures. Low compliance group included 54 patients who did not follow all instructions during treatment period. After the therapy, low compliance patients and their parents were suggested to visit the voiding school.

Voiding school is a measure of specific urotherapy, usually implemented in case of standard urotherapy failure. Unlike standard, specific urotherapy is intensive, focused on small groups of children with treatment-resistant symptoms and combined with pharmacotherapy, if indicated [9]. The voiding school included two 1-day group visits, 6 h each, 3 months apart, and was led by two urotherapists. Children were divided into groups of four to six participants according to their age and sex. The first voiding school visit began with a short discussion with parent to assess the child’s status. When all children arrived and parents left, the children were encouraged to talk about voiding problems. During the first visit, the children were educated on primary MNE and its treatment, by talking, watching videos, drawing, gluing pictures, etc. The children were instructed to go to the toilet regularly at certain times and to drink 1.5 L daily i.e. five glasses of water and three glasses of milk, to avoid juices, fruit syrups and soft drinks, and not to drink more than one glass of water after six o’clock in the evening. Children were advised to try to defecate daily after some meals, to practice an adequate and relaxed toilet posture with the help of a little bench under the feet, not to hurry to leave the toilet, but to count to 10 after voiding. Before the lunchtime children were educated to choose plates with wholesome food. Each child also made his or her own timetable where they marked when it is time to go to the toilet and drink. They learned to follow this timetable both in school and at home. At the end of the visit each child, parent and urotherapist discussed together to provide more personalized approach. The second voiding school visit had a similar basic structure, but the content of the education was carried out based on the children’s individual learning needs and questions, and differed somewhat in each group. The day started with a discussion about urinary tract function and central points of treatment to discover the children’s learning needs. The children were asked to talk what they have learned and done differently after the first voiding school day, and whether they noticed any changes in wetting episodes. They were encouraged to ask questions. The learning methods used were based on children’s learning needs and questions. All children were interviewed in their respective groups at the end of the second visit, in familiar surroundings, by both urotherapists who led the voiding school. During the interviews, the children were encouraged to talk about primary MNE and share their experiences, even if felt embarrassing. The interview guide was used to direct the conversation if necessary (Table 1). Otherwise, the interviewers attempted not to interfere with the conversation. Each interview lasted about 30 min. At the end of the voiding school parents were given a phone appointment for 3 months later, after which the effects of voiding school were assessed. Parents were instructed to contact the urotherapist if something concerning the child distress them at home. Background information was collected from the referrals and discussions with the children and their parents. During the 3 months of voiding school, between first and second day and 3 months after voiding school patients received Desmopressin therapy. Voiding school was attended by 31 child, and all finished the program. Three months later the therapeutic effects of Desmopressin and compliance with therapy were assessed in the identical manner as before school attendance, and then compared with pre-voiding school results.

**Table 1:** The interview guide

1) What things did the children remember from voiding school?	
2) Was voiding school useful according to the children’s opinions?	
3) Have voiding accidents been reduced?	
4) What have the children learned?	
5) In what ways have the learned things appeared at home, in school and during hobbies?	
6) Has incontinence prevented children from doing something?	
7) Have there been any bad situations because of wetting?	
8) How do the children feel about meeting other children with incontinence?	
9) Would the children come to voiding school again?	

Patients were informed about the medication side effects including headache, vomiting, dizziness, edema, hyponatremia, and seizures. None of these side effects were observed.

### Statistical analysis

Categorical variables are presented as absolute numbers with percentages. Numeric variables were presented as means with standard deviations or medians with 25th -75th percentile according to data distribution. Normality was assessed by Kolmogorov-Smirnov test and graphical methods. Enuresis frequency change (before and after therapy) was tested by the Wilcoxon signed-rank test. Percent enuresis reduction was calculated as: % decrease = ((enuresis frequency before-enuresis frequency after)/enuresis frequency before)*100. Differences between low and good compliance groups were analysed by Students t-test or Mann Whitney test, as appropriate. Differences in compliance before and after voiding school was tested with the McNemar test. Violin plots were used to present differences in examined variables according to study groups [10]. Changes in examined variables are presented by line graph where the lines represent summary measure (median) and shaded areas represent interquartile range [11]. The level of significance was set at 0.05. Statistical analysis was performed using the IBM SPSS 21 (Chicago, IL, 2012) package.

## Results

**Fig. 1:**
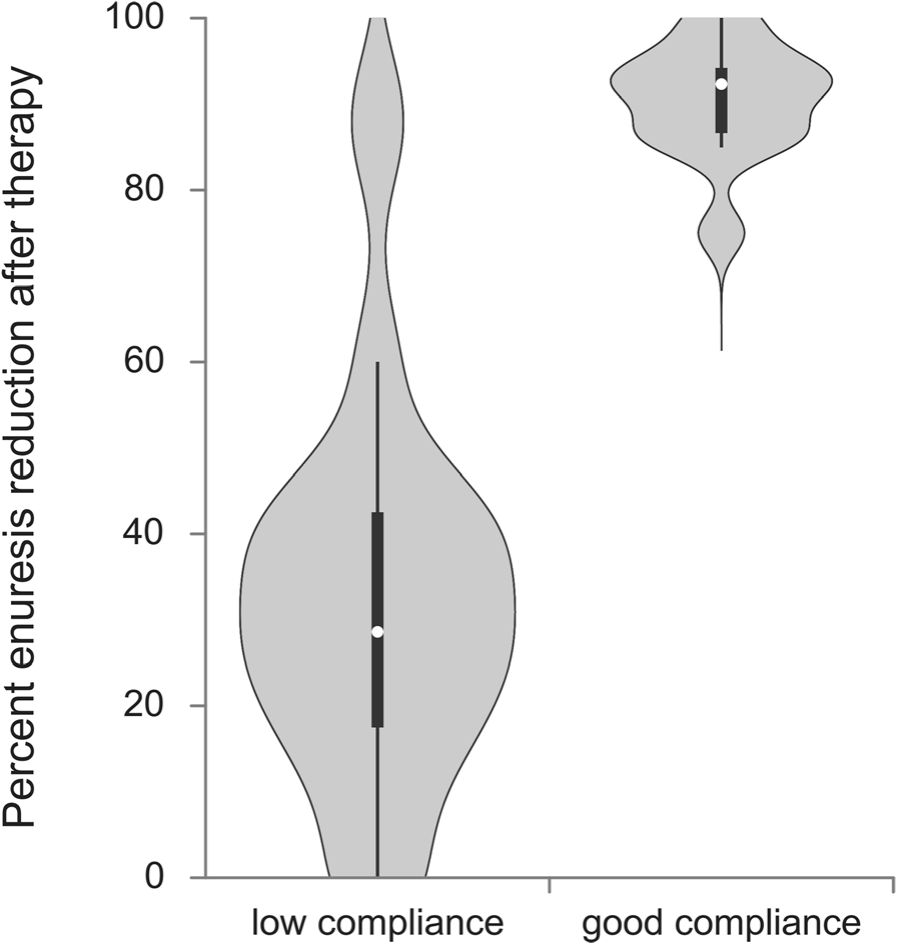
Percent enuresis reduction after therapy in groups with good and low compliance

**Fig. 2:**
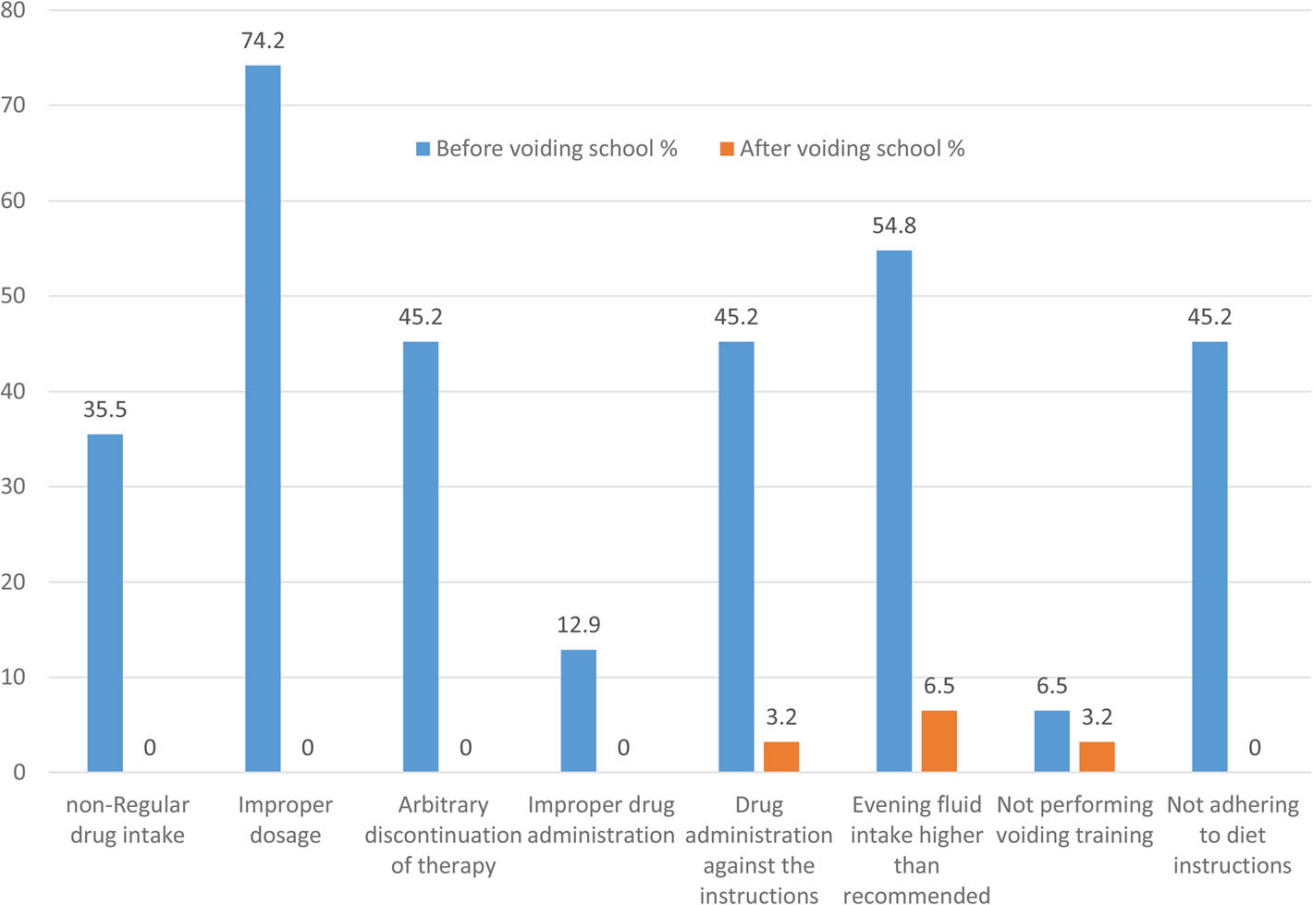
The improvement of compliance after administered voiding school

**Fig. 3:**
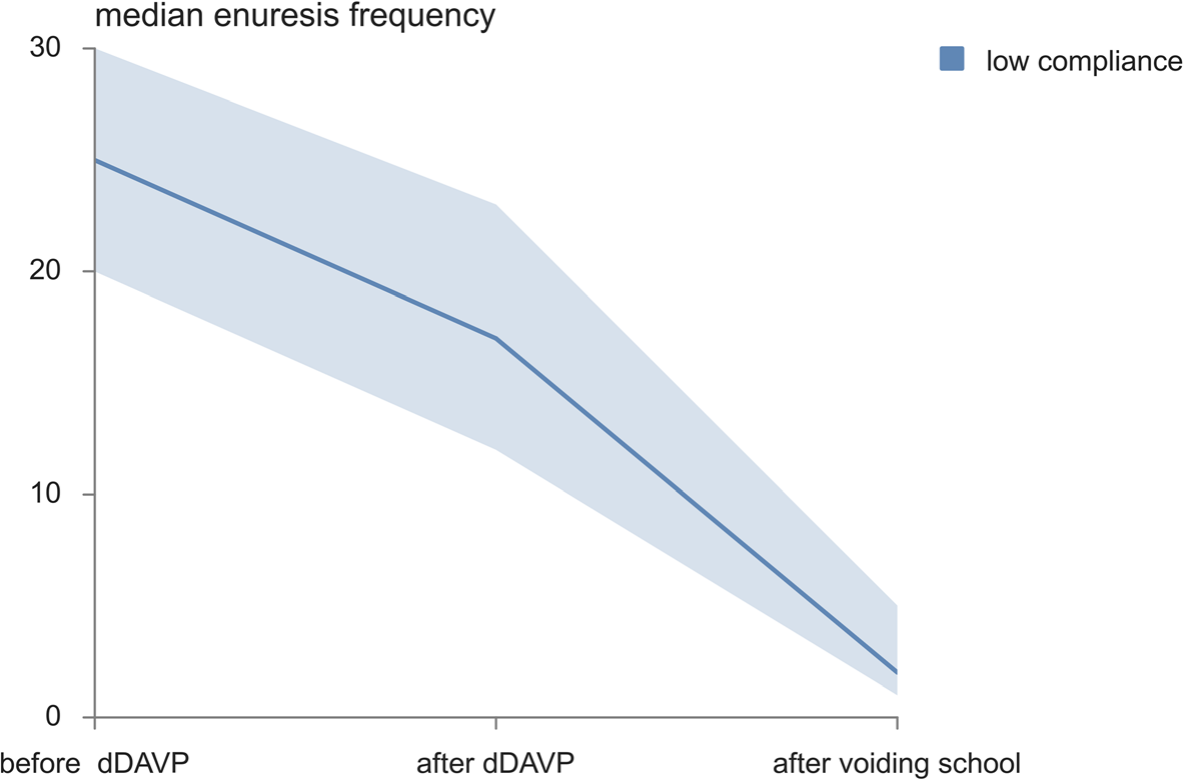
The median enuresis frequency in group with low compliance before and after after voiding school

The study population included 89 patients with primary MNE, out of whom 65 (73%) were boys and 24 (27%) were girls. The average age was 7.7 ± 2.4 years. During the Desmopressin treatment a significant decrease in the enuresis frequency was noticed (*p* < 0.001). Before therapy, a median monthly number of wet nights was 25.0 (20.0–26.0), whereas the monthly median number of wet nights after therapy was 10.0 (2.0–17.0). In good compliance group (*n* = 35) the median percent decrease in enuresis frequency was 92.3 (86.7–95) (Table 2). In low compliance group (*n* = 54) the median percent decrease in enuresis frequency was 28.6 (16.7–43.3) (Table 2). Patients with low compliance had a weaker response to Desmopressin therapy (*p* < 0.001) (Fig. 1). Good- and low- compliance groups did not differ according to gender. In addition to overall compliance, adherence to each separate drug intake or conservative measure instruction was also analyzed in terms of nocturnal enuresis change (Table 2) and significant differences in the therapeutic effects of Desmopressin were noticed with respect to adherence to each separate measure. In addition, a significant increase in compliance was reported among 31 patients in low compliance group who attended voiding school (*p* < 0.001) (Fig. 2). In this group, a bedwetting was reduced for 84.0% (75.0–95.5%) (*p* < 0.001). The median number of bedwetting for low compliance children before and after attending voiding school is shown in Fig. 3. At the end of the study, overall wet night reduction was 43% (26.7–88.0%). In total, 22 (24%) patients showed reduction of wet nights > 90%.

**Table 2:** Percent enuresis reduction according to compliance

		n (%)	Percent enuresis reduction median (25th–75th percentile)	p
Compliance overall	no	54 (60.7)	28.6 (16.7–43.3)	< 0.001
	yes^a^	35 (39.3)	92.3 (86.7–95)	
Non-regular drug intake	no	59 (66.3)	86.7 (43.8–92.3)	< 0.001
	yes	30 (33.7)	20 (12–28)	
Improper dosage	no	76 (85.4)	75 (28.6–91.2)	< 0.001
	yes	13 (14.6)	23.3 (16.7–30)	
Arbitrary discontinuation of therapy	no	67 (75.3)	85 (40–92.3)	< 0.001
	yes	22 (24.7)	16.7 (0–26.7)	
Inproper drug administration	no	45 (50.6)	88 (85–93.3)	< 0.001
	yes	44 (49.4)	28 (16.7–40)	
Fluid intake after the drug intake	no	62 (69.7)	86.7 (28.6–92.3)	< 0.001
	yes	27 (30.3)	30 (20–40)	
Evening fluid intake higher than recommended	no	39 (43.8)	90 (86.7–93.3)	< 0.001
	yes	50 (56.2)	28 (16.7–40)	
Not performing voiding training	no	45 (50.6)	88 (85–93.3)	< 0.001
	yes	44 (49.4)	28 (16.7–40)	
Not adhering to diet instructions	no	61 (68.5)	86.7 (40–92.3)	< 0.001
	yes	28 (31.5)	21.7 (16.7–35)	

^a^Good compliance patients i.e. patients that followed all given instructions (regarding drug intake and other specified conservative measures)

## Discussion

Studies have showed that more than half of children with primary MNE have only 50% decrease in wet nights after Desmopressin treatment. Full response for Desmopressin, defined as > 90% decrease in wet nights, has only been achieved in 20–30% of patients, while partial response (50–90% decrease in wet nights) has 20–40%. Non-optimal response was attributed to: accompanying bladder dysfunction, pharmacokinetic characteristics and suboptimal dosing of Desmopressin, differences in primary MNE definitions, higher osmotic and sodium excretion as well as nutritional and fluid habits [5].

However, little attention has been given to poor compliance with therapy as a possible cause. In our previous work based on the same cohort of patients with primary MNE we hypothesized that the higher incidence of lower maximum voided volume and/or overactive bladder symptoms and lower compliance in youngest children may explain lower Desmopressin effect [8].

This study was planned accordingly, aiming to assess the therapeutic effect of Desmopressin in treating primary MNE according to children’s compliance with therapy protocol. Study results clearly indicate that the therapeutic effect of Desmopressin is directly influenced by compliance, and that low compliance patients, i.e. those that did adhere to all therapy protocol instructions, had a much poorer response to the Desmopressin. We have also reported the extent to which individual adherence to every separate instruction (non-regular drug intake, improper dosage, arbitrary discontinuation of therapy, inproper drug administration, and not following advices related to voiding training, fluid intake and diet) influences the percent decrease in nocturnal enuresis during the Desmopressin therapy. These results confirmed that adherence to all instructions from therapeutic protocol is necessary to achieve the best therapeutic effect of Desmopressin, while non-adherence to some or all of the instructions has a far poorer response to Desmopressin. In addition, we concluded that compliance with therapy for primary MNE in our study was extremely low - our patients had good compliance in only 39% (35/89). Attending well-organised voding school considerably contributed to the increase in compliance with therapy and decrease in bedwetting in our study. Although we did not investigate the possible causes of low compliance in our patients, we concluded that for a drug with an effect duration limited to the night after administration, a high degree of compliance is essential to ensure its consistent therapeutic effects.

Our results are supported by current literature, although treatment compliance in children with enuresis remains under-evaluated. Baeyens et al. showed that higher compliance was correlated with better therapeutic outcome after 6 months of therapy. Their study showed 70% average compliance with advices in children with nocturnal enuresis. They highlighted that complete adherence with medication and recommendations for its usage are essential and may change the response to therapy [12]. Van Herzeele et al. stated that after 3 months of treatment compliance was lower by 10–20%, and that at the end of the study there was 71 to 77% fully compliant children. They emphasize that low compliance was associated with poor response which lead to insufficient antienuretic and antidiuretic effect [5]. Other studies have showed 67% [13] to 94% [14] compliance with Desmopressin.

It is important to highlight that low compliance were more frequent in studies with rigorous definitions of compliance, and that in some studies definitions were not reported. A difference in compliance definitions and sample sizes makes a comparison between studies challenging. One of the reasons for low compliance may be that PNME is not severe and life threatening disease. The current literature suggests various theories for low compliance in primary MNE. Babwah et al. examined compliance in diabetic patients and suggested that women have greater compliance when compared to men [15]. Other demographic factors, such as socioeconomic status and teen age can contribute to low compliance to medical treatment [16]. Also, if patient or their parents perceive enuresis as issue that need and can resolve, they will have more interest to commit fully to treatment. Response to treatment is increasing with higher compliance which further strengthens compliance continuation, but when the response is low - there is further deterioration in compliance. Parents support as well as patients and parents education on primary MNE can contribute to higher compliance due to its effect on children’s psychological condition [17]. Highly compliant children have an advanced problem solving competence and higher self-esteem [12]. Non-optimal dosing and treatment side effects can have negative influence on compliance due to lower treatment effectiveness or acceptability.

Voiding school or bladder training in limited group of children can increase drug treatment results in non responsive cases [6, 7]. Education in similar age groups can influence social competences and children’s acceptance [18, 19]. Saarikovsky et al. reported an improvement after voiding school (60% of children with daytime incontinence, and 50% of children with enuresis showed > 50% decrease in wetting episodes, and 22% children became completely dry) [20]. In contrast, Ferrara et al. found that the effect of motivational therapy associated with Desmopressin on wet nights were enhanced, but not statistically significant [21]. Less promising results of voiding school in current literature might be explained by: different programs of voiding schools, different selection of patients, not recognizing enuresis as a problem, i.e. in the absence of motivation to solve the enuresis, and not using Desmopressin during the voiding school.

High percentage of response after voiding school in our study can be explained by strict selection of patients, as we did not include patients with neurologic and psychiatric comorbidities. Also, high motivation of children and parents to resolve the problem of enuresis in our patient group, as well as the combination of voiding school with Desmopressin may explain our good results. However, there are studies doubting the effects of standard urotherapy that emphasize unclear effect of conservative modalities on the response to Desmopressin, and analyze separately the effects of drug administration management from those of conservative modalities [22, 23].

The limitation of this study is a small sample size and lack of long term follow up needed to verify the persistence of the effects of improved compliance. In this study, we did not determined the specific reasons for low compliance in our patients, which might be a further limitation of the study. Our findings should be expanded in further studies by: including a larger patient population, defining unique definition of compliance, as well as evaluating the specific causes of low compliance, in order to define additional measures that should be taken to achieve maximum therapeutic effect of Desmopressin in treating children with primary MNE.

## Conclusion

Compliance considerably influences the beneficial effects of Desmopressin. Patients with poor therapeutic effects should be evaluated for compliance and introduced to voiding school. Voiding school attendance may have beneficial effects on compliance, which in turn can optimize Desmopressin therapeutic effects to reduce enuresis in children with primary MNE.

## Data Availability

The datasets used and/or analyzed during the current study available from the corresponding author on reasonable request.

## Abbreviations

MNE: Monosymptomatic nocturnal enuresis
NMNE: Non-mono symptomatic nocturnal enuresis
dDAVP: Desmopressin
